# *BCL11A* gene DNA methylation contributes to the risk of type 2 diabetes in males

**DOI:** 10.3892/etm.2014.1783

**Published:** 2014-06-12

**Authors:** LINLIN TANG, LINGYAN WANG, HUADAN YE, XUTING XU, QINGXIAO HONG, HONGWEI WANG, LEITING XU, SHIZHONG BU, LINA ZHANG, JIA CHENG, PANPAN LIU, MENG YE, YIFENG MAI, SHIWEI DUAN

**Affiliations:** 1 Zhejiang Provincial Key Laboratory of Pathophysiology, Ningbo University, Ningbo, Zhejiang 315211, P.R. China; 2 The Affiliated Hospital, School of Medicine, Ningbo University, Ningbo, Zhejiang 315000, P.R. China; 3 Bank of Blood Products, Ningbo No. 2 Hospital, Ningbo, Zhejiang 315010, P.R. China; 4 Section of Endocrinology, Pritzker School of Medicine, University of Chicago, Chicago, IL 60637, USA

**Keywords:** type 2 diabetes, *BCL11A*, DNA methylation, CpG island, gender

## Abstract

*BCL11A* is a critical modulator involved in hemoglobin switching. Recent studies have established an association between *BCL11A* gene polymorphisms and a risk of type 2 diabetes (T2D). The aim of the present study was to assess the correlation between *BCL11A* DNA methylation and T2D. A total of 48 T2D cases and 48 age- and gender-matched controls were recruited to evaluate *BCL11A* methylation using bisulfite pyrosequencing technology. Although no significant association was observed in *BCL11A* methylation between T2D patients and healthy controls (P=0.322), breakdown analysis by gender identified a significant association between *BCL11A* methylation and T2D in males (P=0.018). Notably, there was also a significant female-specific association between the mean *BCL11A* DNA methylation and triglyceride (TG) concentration (r=−0.34; P=0.019). The results indicated that *BCL11A* methylation contributed to the risk of T2D in males. In addition, *BCL11A* methylation may have an effect on the development of T2D by influencing TG metabolism. Thus, gender difference may provide new information to aid the understanding of T2D pathogenesis.

## Introduction

Type 2 diabetes (T2D) is a chronic disease that affects glucose metabolism. T2D has a number of associated serious complications, including heart disease, retinopathy and renal failure (1,2). The prevalence of diabetes mellitus, particularly T2D, is increasing with contributing factors, such as body weight and obesity (3). The T2D population is predicted to be double the size as it is currently by 2030 (4). T2D is affected by genetic (5) and environmental factors, including an unhealthy lifestyle (6), diet (7) and obesity (8).

Although there are hundreds of genetic loci associated with T2D (9), >90% of T2D trait variations remain to be explained. Epigenetic modification, including DNA methylation, plays an important role in the pathogenesis of T2D (10). DNA methylation and histone modification have become alternative approaches that have aided the understanding of β-cell dysfunction in the pathogenesis (11) and the high growth rate of T2D (12). Aberrant DNA methylation of genes, such as PGC-1α (13), *PDX1* (14), MCP-1 (15) and leptin (16), have been shown to contribute to the risk of T2D. In addition, a number of environmental risk factors, including malnutrition and a lack of physical exercise, interfere with DNA methylation modification and increase the risk of T2D (17).

The *BCL11A* gene encodes a CH2H2 type zinc-finger protein that is necessary for lymphopoiesis and the negative regulation of p53 activity (18), functioning as a transcriptional repressor (19). Elevated levels of insulin and leptin and decreased levels of adiponectin in the serum are known to be associated with T2D risk, and they may also downregulate p53 expression,thus, induce a cancer risk (20). Expression of human fetal hemoglobin is controlled by *BCL11A* (21). *BCL11A* deficiency is associated with decreased fetal hemoglobin (22), which is significantly associated with a decreased risk of T2D (23). *BCL11A* gene variants affect the insulin response to glucose (24) and glucagon secretion (25), thus, have been shown to increase the risk of T2D in Europeans, North African Arabs (26) and African-Americans (27). The aim of the present study was to investigate the contribution of *BCL11A* DNA methylation to the risk of T2D.

## Materials and methods

### Sample collection

A total of 48 T2D cases and 48 age- and gender-matched controls were selected from patients in the Affiliated Hospital of Ningbo University and Ningbo No. 2 Hospital (Ningbo, China). Patients were included in the study if they met the following criteria. Firstly, all the subjects were recruited without hypertension, coronary heart disease or other serious diseases. Secondly, the subjects were of Han Chinese origin and had lived in Ningbo city for at least three generations. Thirdly, standard clinical criteria (World Health Organization, 2007; 28) were applied with regard to T2D diagnosis, while the selection for healthy controls was based on the standard that the fasting blood glucose level was <6.1 mmol/l. Blood samples were collected from all the participants and were stored at −80°C in 3.2% citrate sodium-treated tubes. All the involved individuals provided informed consent, which was approved by the Ethical Committees of the Affiliated Hospital of Ningbo University and Ningbo No. 2 Hospital.

### Phenotype and biochemical analyses

Phenotype analysis included total cholesterol (TC), triglyceride (TG), alanine aminotransferase (ALT), uric acid (UA) and glucose levels. Plasma levels of TG and TC were measured using an enzymatic end point assay (29). Concentrations of ALT and blood glucose were measured using the International Federation of Clinical Chemistry reference measurement systems (30) and the glucose oxidase and peroxidase assay (31), respectively. UA levels were measured with a CX77 Analyzer (Beckman Coulter, Inc., Fullerton, CA, USA). Genomic DNA was isolated from peripheral blood samples using a nucleic acid extraction analyzer (Lab-Aid 820; Xiamen City, China), and the concentration was measured using a NanoDrop 1000 spectrophotometer (Thermo Fisher Scientific, Wilmington, DE, USA). *BCL11A* methylation was conducted with pyrosequencing technology combined with sodium bisulfite DNA conversion chemistry (EpiTech Bisulfite kits; Qiagen, Venlo, Netherlands) and polymerase chain reaction (PCR) amplification (Pyromark PCR kit; Qiagen). PyroMark Assay Design software automatically selected the appropriate CpG sites with high scores in a 70-nt fragment to design the PCR and sequencing primers, which included the forward (5′-GTTTAGGTTAGAGGTGGGTGTTT-3′), reverse (5′-biotin-TATACCAATCTTCTCCTTACTACCT-3′) and sequencing primers (5′-GAAGGGTAGGAGTTA-3′). The biotin in reverse primer was used to identify the sequences.

### Statistical analysis

SPSS software (version 16.0; SPSS, Inc., Chicago, IL, USA) was used for all the statistical tests, including the t-test for two independent samples, two-way analysis of variance (ANOVA) and Pearson’s regression analysis. Using the two independent samples t-test, *BCL11A* methylation and other phenotypes were compared between the T2D cases and controls. The interaction between gender and T2D status was assessed by applying two-way ANOVA, while the correlation analyses between *BCL11A* methylation and other phenotypes (including TG, TC, UA and ALT) were performed with Pearson’s regression analysis. P<0.05 was considered to indicate a statistically significant difference.

## Results

### Association between mean BCL11A methylation and T2D

**Figure 1:**
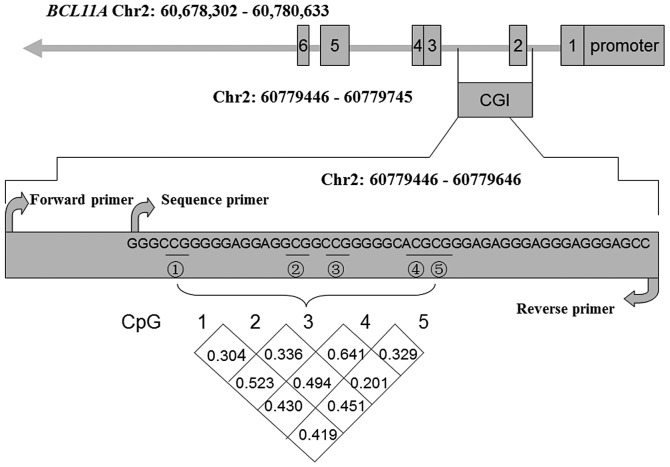
Pairwise correlation of the methylation of the five CpGs on *BCL11A*.

**Figure 2:**
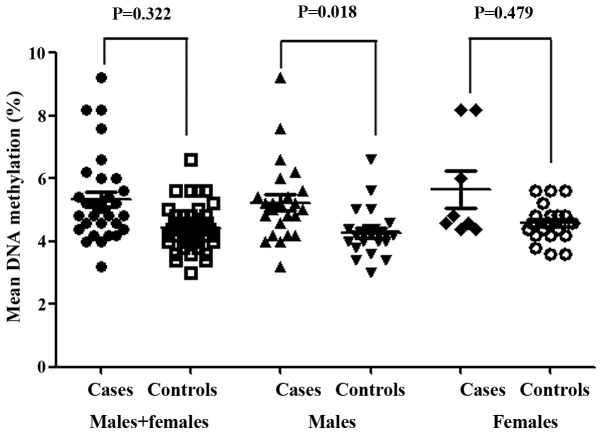
Comparison of the mean DNA methylation levels between T2D cases and controls. T2D, type 2 diabetes.

As shown in Fig. 1, the intragenic CpG island (CGI) was close to the promoter. A total of five CGI sites that exhibited a strong correlation were used to evaluate the DNA methylation of the *BCL11A* gene (r>0.3; Fig. 1). Although there was no statistically significant gender difference with regard to mean DNA methylation (Table I; P=0.102), a significant difference in the mean DNA methylation of the *BCL11A* gene was observed in males (Fig. 2; P=0.018).

### Association between mean BCL11A methylation and clinical phenotypes

**Figure 3:**
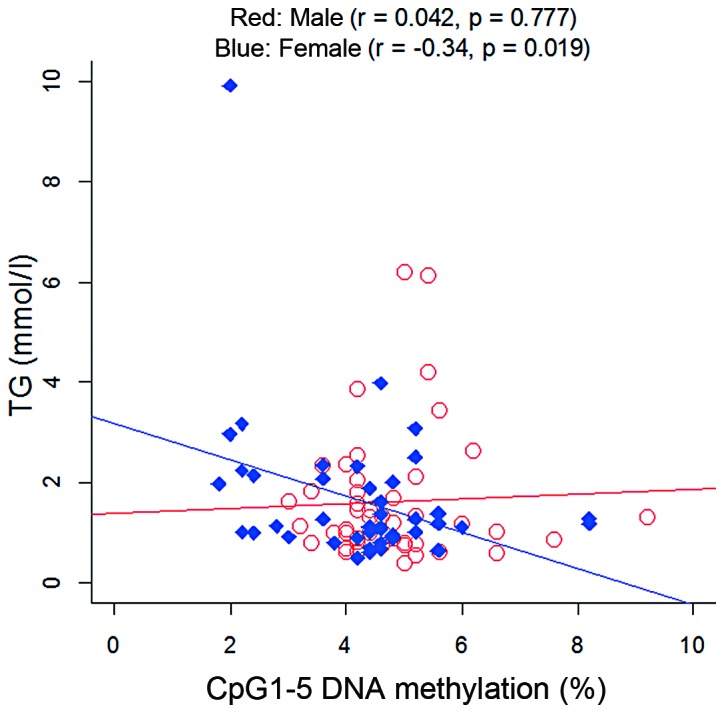
Correlation between the mean *BCL11A* DNA methylation and TG levels. TG, triglyercide.

As shown in Table I, among the five tested phenotypes, the results demonstrated that TC (P=0.021), ALT (P=0.019) and UA (P<0.001) levels were significantly different between males and females, and that levels of TG (P=0.038) and ALT (P=0.006) were significantly different between the T2D cases and controls. The results also revealed a significant interaction between gender and T2D status for the association study of mean *BCL11A* methylation (P=0.003). In addition, a female-specific correlation between the TG level and mean DNA methylation was observed (Fig. 3; P=0.019). However, no correlations were observed between the other phenotypes (age, TC, ALT and UA) and mean DNA methylation (P>0.05).

## Discussion

In recent years, an increasing number of studies have investigated DNA methylation in a variety of diseases, including coronary heart disease (32), lung cancer (33) and T2D (34). The present study investigated the association between *BCL11A* DNA methylation and the risk of T2D in 48 T2D cases and 48 age- and gender-matched controls. The results revealed that *BCL11A* DNA methylation was specifically associated with the risk of T2D in males (P=0.018).

A previous study demonstrated gender differences in T2D (35). Compared with males, a higher prevalence for cardiovascular disease was shown in diabetic females (36). Female T2D patients have compact clots and compromised fibrinolysis, thus, are much more likely to suffer from atherothrombotic disease (37) compared with male T2D patients. In addition, serum ferritin levels have been shown to be significantly associated with fasting glucose levels in female T2D patients (38). Gender differences have also been observed in the association between other diseases and the methylation of genes, including *PLA2G7* (32), *MIR375* (34) and *MTHFR* (39). The present study demonstrated a male specific association between *BCL11A* DNA methylation and the risk of T2D, but a female-specific correlation between TG levels and DNA methylation.

High TG/high-density lipoprotein cholesterol levels are associated with the risk of microvascular complications in T2D (40). Continuous insulin infusion can correct hypertriglyceridemia in T2D patients and markedly reduce the risk of metabolic complications (41). The development of T2D may be associated with DNA methylation in the *BCL11A* gene via affecting TG levels.

CGIs in the promoter regions of diabetic candidate genes, such as *MIR375* (34), are associated with the risk of T2D. Although DNA methylation of gene promoters has a significant impact on gene expression, a correlation exists between intragenic DNA methylation and gene expression (42). The present study demonstrated that intragenic DNA methylation in the *BCL11A* gene was associated with T2D. However, there were limitations to the study. For example, the sample size of the study was relatively small, which should be expanded for future study. In addition, DNA methylation is tissue specific and the observations in the peripheral blood may not reflect the other tissues of interest.

In conclusion, the present study revealed a male-specific significant association between *BCL11A* DNA methylation and the risk of T2D and a female-specific association between TG levels and and *BCL11A* DNA methylation. These observations may improve the understanding of the molecular mechanisms underlying T2D pathogenesis.

## Figures and Tables

**Table I:** Characteristics of the subjects.

Characteristics	Males (n=47)	Females (n=47)^b^	P1	Diabetic (n=48)	Non-diabetic (n=46)	P2	P3
Age (years)	59.0±8.7	59.2±6.2	0.902	59.2±7.5	58.9±7.5	0.839	0.948
TC (mmol/l)	4.96±0.95	5.42±0.93	0.021	5.34±0.83	5.04±1.08	0.133	0.037
TG (mmol/l)	1.60±1.29	1.62±1.46	0.965	1.90±1.69	1.32±0.84	0.038	0.546
ALT (mmol/l)	26.64±19.22	17.38±7.42	0.019	25.10±18.49	16.74±8.23	0.006	0.236
UA (μmol/l)	323.24±78.08	263.56±72.24	<0.001	289.30±70.48	297.67±90.57	0.065	0.058
DNA methylation (%)^a^							
CpG1	5.89±1.784	5.447±2.224	0.334	5.98±2.46	5.35±1.37	0.076	0.094
CpG2	2.319±1.520	2.213±1.587	0.705	2.56±1.60	1.96±1.44	0.036	0.012
CpG3	7.234±1.902	6.298±2.255	0.107	6.96±2.63	6.57±1.44	0.427	0.019
CpG4	6.809±1.262	6.021±2.101	0.011	6.17±2.26	6.67±0.99	0.318	0.002
CpG5	1.575±1.315	1.511±1.040	0.935	1.46±1.20	1.63±1.16	0.592	0.264
Mean	4.766±1.139	4.298±1.373	0.102	4.63±1.66	4.43±0.67	0.322	0.003

a DNA methylation in the T2D cases and controls were adjusted by TG and ALT.

b Two female samples were excluded due to repetitive failures in the methylation sequencing. Results are expressed as the mean ± standard deviation.

TC, total cholesterol; TG, triglyceride; ALT, alanine aminotransferase; UA, uric acid; T2D, type 2 diabetes. P1, P-value between males and females; P2, P-value between diabetic samples and non-diabetic samples and P3, the interaction P-value between gender and T2D status

## Abbreviations

T2D: type 2 diabetes
TC: total cholesterol
TG: triglyceride
ALT: alanine aminotransferase
UA: uric acid
CGI: CpG island
